# Profile and Functional Properties of Seed Proteins from Six Pea (*Pisum sativum*) Genotypes

**DOI:** 10.3390/ijms11124973

**Published:** 2010-12-03

**Authors:** Miroljub Barac, Slavica Cabrilo, Mirjana Pesic, Sladjana Stanojevic, Sladjana Zilic, Ognjen Macej, Nikola Ristic

**Affiliations:** 1 Faculty of Agriculture, University of Belgrade, Nemanjina 6, 11000 Belgrade-Zemun, Serbia; E-Mails: mpesic@agrif.bg.ac.rs (M.P.); sladjas@agrif.bg.ac.rs (S.S.); macej@agrif.bg.ac.rs (O.M.); risticn@agrif.bg.ac.rs (N.R.); 2 High Technical School of Vocational Studies, Pozarevac, Nemanjina 2, Serbia; E-Mail: slavica.cab@sbb.rs; 3 Maize Research Institute, “Zemun Polje”, Slobodana Bajića 1, 11000 Belgrade-Zemun, Serbia; E-Mail: szilic@mrizp.rs

**Keywords:** pea proteins, extractability, emulsifying, foaming

## Abstract

Extractability, extractable protein compositions, technological-functional properties of pea (*Pisum sativum*) proteins from six genotypes grown in Serbia were investigated. Also, the relationship between these characteristics was presented. Investigated genotypes showed significant differences in storage protein content, composition and extractability. The ratio of vicilin:legumin concentrations, as well as the ratio of vicilin + convicilin: Legumin concentrations were positively correlated with extractability. Our data suggest that the higher level of vicilin and/or a lower level of legumin have a positive influence on protein extractability. The emulsion activity index (*EAI*) was strongly and positively correlated with the solubility, while no significant correlation was found between emulsion stability (*ESI*) and solubility, nor between foaming properties and solubility. No association was evident between *ESI* and *EAI*. A moderate positive correlation between emulsion stability and foam capacity was observed. Proteins from the investigated genotypes expressed significantly different emulsifying properties and foam capacity at different pH values, whereas low foam stability was detected. It appears that genotype has considerable influence on content, composition and technological-functional properties of pea bean proteins. This fact can be very useful for food scientists in efforts to improve the quality of peas and pea protein products.

## 1. Introduction

Peas (*Pisum sativum* L.) are becoming an important vegetable source of proteins and a potential alternative to soybean in Europe. The increased acceptance of pea proteins is due to pea manifold qualities, good functional properties in food applications, high nutritional value, availability, and relatively low cost. Also, pea beans and their products are a rich source of biologically active components that may exert beneficial health and therapeutic effects [1].

The major pea storage proteins referred as legumin (11S), vicilin (7S) and convicilin are globulins. Pea legumin is hexamer with a molecular weight (Mw) ∼320 to 380 kDa. The mature proteins consist of six subunit pairs that interact noncovalently. Each of these subunit pairs consists, in turn, of an acidic subunit of ∼40 kDa and a basic subunit of ∼20 kDa, linked by a single disulfide bond [2]. As there are a number of legumin precursors originating from several gene families, different legumin polypeptides have been identified, e.g., 4–5 acidic (α) and 5–6 basic (β) polypeptides [3]. The sizes of these polypeptides range from 38–40 kDa for the acidic polypeptides, and from 19–22 kDa for the basic polypeptides. Vicilin is a trimeric protein of ∼170 kDa that lacks cysteine residues and hence cannot form disulfide bonds [4]. Subunits composition of pea vicilin varies mostly because of post-translation processing. Mainly, vicilin consists of ∼47 kDa, ∼50 kDa, ∼34 kDa and ∼30 kDa subunits [5]. A third major storage protein, named convicilin, has a subunit of ∼71,000 and a molecular weight in its native form of 290 kDa [6]. O’Kane [7] denoted this protein as α-subunits of vicilin. The ratio of vicilin to legumin varies among genotypes and may range from 0.5 to 1.7, with a mean of 1.1 [8].

The differences in content, composition and structure between vicilin and legumin are exhibited in both nutritional and functional properties. Legumin contains more sulfur containing amino acids than vicilin per unit of protein [4], and its more available fraction from a nutritional point. Furthermore, different functional properties of these proteins have been reported. It was found by Bora *et al.* [9] that pea vicilin underwent heat-induced gelation, whereas legumin did not gel under the same conditions. O’Kane *et al.* [10] indicated that both pea vicilin and legumin could form gels. These authors [11] showed that the contribution of legumin to the pea protein gels was cultivar specific. Also, vicilin was shown to possess better emulsifying properties than legumin [12–14]. Technological-functional properties of pea protein-based products depend on several factors including protein content and composition of starting pea bean, the purification and processing method. Protein content varies among genotypes [15–17] and is influenced by environmental factors [17,18]. Maninder Kaur *et al.* [19] investigated the functional properties of flours from two field pea varieties. They reported significantly different emulsifying, foaming properties, as well as water and oil holding capacity between flour prepared from these varieties. Several studies [20–22] based on soy proteins were carried out to establish the relationship between protein composition and functional properties. Pešić *et al.* [22]examined twelve soybean genotypes and reported that soybean variety had significant effect on the 11S:7S protein ratio of soybean seed. They showed that the emulsion properties, such as emulsion activity, were highly correlated with 11S:7S ratio. Furthermore, the purification, as well as processing may have an influence on protein composition of obtained protein product, which reflects on functional properties [23–27].

The aim of this research is to contribute to understanding the influence of genotypes on the composition and technological-functional properties of pea proteins. The present study is composed of two parts. The first characterizes the protein profiles of three commonly grown cultivars of field pea in Serbia and three experimental lines. The objective of the second part of our investigation was to isolate pea proteins from selected pea grains by isoelectric precipitation (pI), to characterize their protein compositions and their functional properties.

## 2. Experimental Section

### 2.1. Material

Six pea genotypes, three varieties: Maja, Calvedon, Miracle of America commonly grown in Serbia, and three experimental lines (L1, L2 and L3) grown in 2009, in field conditions were investigated. L1, L2 and L3 were high seed-protein lines selected by the Institute of Field and Vegetable Crops (Smederevska Palanka, Serbia). Commercial pea protein isolate (Pisane™, Cosucra, Belgium) was a gift from Kuk d.o. (Belgrade).

Pea protein isolate was obtained by isoelectric precipitation of dry pea meal as reported by Sumner *et al.* [28], with minor modifications. Dried pea seed was ground in a home mixer (Fisher, Germany). 50 g portions of the flour were dispersed in 500 mL of MiliQ water and stirred for 15 min to obtain uniform dispersions. The pH of the obtained suspensions was adjusted with 1 mol dm^3^ NaOH to pH 9.0, stirred for 1 hour at room temperature and centrifuged (4,000 × g for 10 min) to remove insoluble components such as fibers. Supernatant was separated, and the insoluble part is re-extracted for 30 min at pH 9.0 and centrifuged again. Supernatant was merged and then precipitated at pH 4.5, stored for 2 hours at 4 °C and centrifuged (4,000 × g for 15 min). Precipitate was re-dissolved during 30 min at pH 9.0, precipitated at pH 4.5, centrifuged and the insoluble fraction (representing the protein isolate) was collected, re-dissolved at pH 7.0 and lyophilized.

### 2.2. Protein Content Determination

The total protein content was determined by the micro-Kjeldahl method using nitrogen to protein conversion factor of 6.25. To determine the extractable flour protein content, the protein was extracted according to the method of Thanh and Shibasaki [29]. The pea flour (1 g) was extracted for 120 min at room temperature with Tris-HCl buffer pH 8.0 and Milli-Q water adjusted to pH 8.0 (the sample to buffer and sample to water to ratio was 1:20) and was centrifuged at 1,7000 × g for 15 min. The protein content in the supernatant was determined according to the method of Bradford *et al.* [30] using commercial pea isolate Pisane M (Cosucra, Belgium, total protein content 852.2 g kg^−1^) as a standard. The protein extractability was expressed as g of extractable protein per kg of total protein.

### 2.3. SDS-PAGE

SDS-PAGE was performed according to the procedures of Fling and Gregerson [31] using 50 g kg^−1^ stacking and 125 g kg resolving gel. Prior to electrophoresis, the protein extract was diluted to 2g L^−1^ with sample buffer (0.055 mol L^−1^ Tris-HCl, pH 6.8, 20 g kg^−1^ SDS, 70 g kg^−1^ glycerol, 43 g kg^−1^ β-mercaptoethanol, 0.025 g kg^−1^ bromophenol blue), heated at 90 °C for 2 min and cooled at room temperature. A 25 μL sample was loaded per well. The gels were run at 30 mA per gel for 6 hours. Gels were fixed, stained with 2.3 g kg^−1^ Coomassie Blue R-250 [dissolved in 39 g kg^−1^ trichloroacetic acid (TCA), 60 g kg^−1^ acetic acid, and 170 g kg^−1^ methanol] for 45 min and destained with 80 g kg^−1^ acetic acid and 180 g kg^−1^ ethanol. Molecular weights of the polypeptides were estimated by using low molecular weight calibration kit (Pharmacia, Sweden). Molecular weight markers included: phosphorylase B (94.0 kDa), bovine albumin (67.0 kDa), ovalbumin (43.0 kDa), carbonic anhydrase (30.0 kDa), soybean trypsin inhibitor (20.1 kDa), and α-laktalbumin (14.4 kDa). Also, the identification was done using 7S and 11S protein fraction obtained by selective isoelectric precipitation. SDS-PAGE was performed with electrophoresis unit LKB-2001-100 in conjunction with power supply LKB-Macrodrive 5 and LKB-MultiTemp as a cooling unit (LKB, Sweden).

SDS-electrophoresis of pea bean and isolated proteins was performed in duplicate. Namely, two aliquots of the same sample were analyzed at the same time. Two gels were run simultaneously in the same electrophoretic cell.

### 2.4. Densitometric Analysis

The destained gels were scanned and analyzed by SigmaGel software version 1.1 (Jandel Scientific, San Rafalel, CA). The determination of vicilin and legumin was made, and their concentrations and ratio were calculated from the sum of the total area of their subunits [32]. Each pattern was analyzed in triplicate.

### 2.5. Protein Solubility

Protein solubility at different pH (3.0; 5.0; 7.0 and 8.0) was determined according to the method of Wu *et al.* [33]. Four 0.020 g-portions of isolate were each dispersed in 20 mL Milli-Q water and stirred for 30 min to obtained uniform dispersions. The pH of suspensions was adjusted with 1 M NaOH or 1 M HCl to pH 3.0; 5.0; 7.0 and 8.0, stirred for 1 hour and centrifuged for 15 min at 17,000 × g (Sigma, Germany). The soluble protein content in the supernatant was determined according to the method of Bradford [30]. Total protein content was determined by extracting 0.020 g of isolate in 20 mL of 0.5 mol dm^3^ NaOH. The protein solubility was expressed as g of soluble protein per kg of total protein.

### 2.6. Emulsifying Properties

Emulsifying properties were measured according to a modified method of Wu *et al.* [33]. Pure sunflower oil (15 mL) and 45 mL 1.0 g kg^−1^ protein isolate suspension, prepared as described for protein solubility determination, were homogenized in a mechanical homogenizer at the highest settings for 1 min. Fifty-micro liter portions of the emulsions were pipetted from the bottom of the container at 0 and 10 min after homogenization. Each portion was diluted with 10 mL of 1 g kg^−1^ SDS solution. Absorbances of these diluted emulsions were measured at 500 nm. The absorbances measured immediately (*A*_0_) and 10 min (*A*_10_) after emulsion formation were used to calculate the emulsifying activity index (*EAI*) and the emulsifying stability index (*ESI*):

(1) $$\text{EAI}({\text{m}}^{2}/\text{g})=2T(A_{0}\times F/C\times \phi \times 10,000)$$

where *T* = 2.303; *A*_0_ = absorbance measured immediately after emulsion formation; dilution factor = 200, *C* = weight of protein/unit volume (g mL^−1^) of aqueous phase before emulsion formation; *Φ* = oil volume fraction of the emulsion; and

(2) $$\text{ESI}(\text{min})=A_{0}\times \Delta t/\Delta A$$

where Δ*t* = 10 min and Δ*A = A*_0_ *− A*_10_.

The *EAI* and *ESI* were measured in two different days, producing each day two different emulsions of the same sample, and taking three aliquots of each emulsion.

### 2.7. Foaming Properties

These properties were expressed as foaming capacity (*FC*) and foam stability (*FS*) according to the method of Sathe and Salunke [34]. Foaming was induced by bubbling a stream of air (6 dm^3^ min^−1^) during 15 s through a Waters filter holder (Waters, U.S.) placed at the bottom of a 250 mL graduated column containing 30 mL of 1g kg^−1^ protein water solution adjusted to pH 3.0, 5.0, 7.0 and 8.0. Foaming properties were expressed as:

(3) FC(%)=A−B/B×100

where *A* = volume of suspension and foam after bubbling, *B* = volume of suspension before bubbling; and

(4) $$\text{FS}(\%)=A_{1}-B/B\times 100$$

where *A*_1_ = volume of suspension and foam after 3 min.

### 2.8. Statistical Analysis

The data were analyzed using Statistica software version 5.0 (StatSoft Co., Tulsa, OK). The significance of differences between means was determined by *t*-test procedure for independent samples at *p* < 0.05. Regression analyses were also carried out.

## 3. Results and Discussion

### 3.1. Extractibility of Pea Bean Proteins

Data in Table 1 indicate that the total protein content, soluble protein concentration, as well as extractability among analyzed pea bean genotypes differ significantly (*p* < 0.05). The exceptions were differences in content of Tris-extracts of genotypes Calvedon, L3 and Maja (147.81 ± 1.2 g kg^−1^; 150.63 ± 0.82 g kg^−1^; 148.65 ± 1.43 g kg^−1^), as well as water extracts of L1 and Maja (117.20 ± 1.01 g kg^−1^; 115.41 ± 0.8 g kg^−1^) that were not statistically significant.

L1 genotype was higher in total protein (317.63 ± 0.29 g kg^−1^) than in the otherones, which varied from 223.31 g/kg (Miracle of America) to 273.70 ± 0.10 g kg^−1^ (Maja). Also, L1 genotype is characterized by the highest content of extractable proteins (227.11 ± 0.62 g kg^−1^, 117.20 ± 1.01 g kg^−1^) in Tris-HCl buffer as well as in water. Very strong positive correlation (0.92, *p* < 0.05) exists between total protein content and content of protein extractable in Tris-buffer. Also, a strong positive correlation (0.88, *p* < 0.05) exists between protein extractability in buffer, as well as in water with adjusted pH and extractable protein content.

According to our results, better extractability is obtained by Tris-HCl buffer pH 8.0 than by water with the same pH value. The average extractability of all genotypes in Tris-buffer and water was about 600 g kg^−1^ and 400 g kg^−1^, respectively. Better extractability of pea bean proteins in Tris-HCl pH 8.0 compare to distilled water with the same pH value, can be attributed to the buffer composition as well as to the tendency of these proteins to form less soluble complexes during water extraction. Namely, Tris-HCl buffer pH 8.0 contained a small amount of 2-mercaptoethanol (0.01 mol L^−1^) and salts, whose presence prevented the formation of less soluble complexes and thus facilitated their extraction. This is in agreement with results reported by Alonso *et al.* [35]. They found that extraction with buffer containing 2-mercaptoethanol (2-ME) or sodium dodecyl sulphate (SDS), alone or in combination, greatly increased protein extractability.

### 3.2. Pea Bean Protein Composition

**Figure 1.**
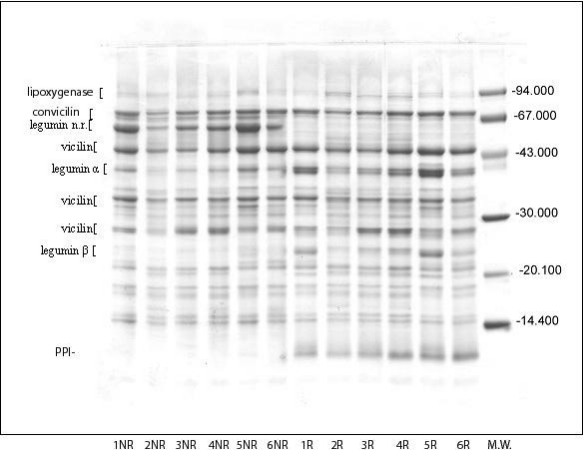
Electrophoretic patterns of pea bean proteins under reducing (R) and non reducing (N.R) conditions. Calvedon (1R, 1NR); L1 (2R, 2NR); L2 (3R, 3NR); L3 (4R, 4NR); Maja (5R, 5NR); Miracle of America (6R, 6NR), M.w. molecular weight standards. Tris-HCl (pH 8.0) extracts with 2-mercaptoethanol (reducing conditions) and without 2-mercaptoethanol (non reducing conditions).

The protein composition of total pea bean proteins as separated on SDS-PAGE under reducing and non-reducing conditions is provided in Figure 1. The concentration of protein subunits is shown in Table 2. SDS-PAGE separated total pea bean proteins into multiple components with M.w. ranging from 104.8 kDa to 9.8 kDa, which originated mainly from vicilin and legumin. The SDS-PAGE patterns of Tris-extracts contained three major (47.3, 35.0, 28.7 kDa) and three minor (37.0, 33.3, 31.8 kDa) subunits of vicilin, as well as two subunits of convicilin (M.w. 77.9 kDa, 72.4 kDa). Legumin was identified with four bands of acidic (M.w. 40.89 kDa) and basic (22.3, 23.1 kDa) subunits. Under reducing condition three minor bands of trimeric (non-reduced) form of legumin (M.w. 63.5 kDa) were detected. Non-reducing conditions promote the reassotiation of legumin subunits into trimeric form registered as intesive band with the same M.w. Also, the minor bands of 92.7 kDa and 11.5 kDa were identified as lypoxigenase (Lox) and protease inhibitor (PPI), respectively. The molecular weight of identified subunits and polypeptides calculated based on the R_f_ value was consistent with the previous work of several authors [5,36,37].

Under reducing conditions, subunits of vicilin, convicilin and legumin were dominant in extracts of all genotypes. Their contents ranged from 80.01% (Maja) to 71.11% (Calvedon) of total extracted proteins. The concentrations of convicilin and vicilin of all genotypes were similar and ranged from 71.6 ± 2.0 g kg^−1^ to 89.8 ± 2.2 g kg^−1^ and from 341.9 ± 1.7 g kg^−1^ to 377.3 ± 1.3 g kg^−1^, respectively (Table 2.). The extracts of L1 had the highest, while Calvedon had the lowest concentration of vicilin. More expressed difference among genotypes in concentration of extracted legumin were registered. The concentration of legumin ranged from 252.4 ± 2.0 g kg^−1^ (L1) to 347.7 ± 2.6 g kg^−1^ (Maja). Even under reducing conditions small part of legumin subunits existed as trimeric form. Trimeric form of legumin ranged from 33.6 ± 1.1 g kg^−1^ (Calvedon) to 75.6 ± 0.3 g kg^−1^ (L1). The ratio of vicilin:legumin varied from 1.06 to 1.49 among the investigated genotypes. Maja had the lowest vicilin:legumin ratios, whereas L1 had the highest. The equal ratio of these proteins were detected in extracts of Calvedon and Miracle of America (1.20) and in extracts of L2 and L3 (1.30, 1.33). Furthermore, the ratio of vicilin + convicilin:legumin ranged from 1.30 (Maja) to 1.78 (L1). A strong positive correlation (0.88, *p* < 0.05) exists between protein extractability and the vicilin:legumin ratio, as well as between extractability and the vicilin + convicilin/legumin ratio. On the other hand, as discussed above, an increase of extractable soluble protein content leads to an increase of protein extractability. These facts indicate that genotypes with a higher level of 7S and/or a lower level of 11S proteins would have higher extractability than others. In addition, pure solutions of vicilin have better functional properties, such as gelling and emulsifying, then pure solutions of legumin. This is probably due to differences in protein structure of 7S and 11 proteins. Thus, genotype with the higher ratio of V/L could be more suitable for protein isolate preparation. However, no correlation was found either for vicilin or legumin concentration with extractability (Table 2), probably because extractability is expressed on the basis of total protein content. Furthermore, our results indicate that some other proteins contribute also to enhancement of protein extractability.

Under non-reducing conditions (Figure 1, line 1NR-6NR) protease inhibitor disappeared almost completely, and it was registered as diffused pale band. This may be as a result either of self-aggregation, or the interactions with subunits of other proteins. It is known that protease inhibitors from BBI family undergo self-aggregation in non-reducing conditions [38]. The concentration of PPI ranged from 1.0 ± 0.3 g kg^−1^ to 9.4 ± 0.1 g kg^−1^ (Table 2). Also, the decrease of dissociated acidic and basic subunits concentration of legumin was perceived. It seems that the part of these subunits re-associates into trimeric form, while another part becomes insoluble. As a result, the ratio of non-reduced/reduced forms of legumin, except in extracts of L1, increased to 0.97–1.22 (Table 2). Also, the ratio of vicilin/legumin increased from 1.28 (Maja) to 2.09 (L1).

### 3.3. Pea Protein Isolates Composition

**Figure 2.**
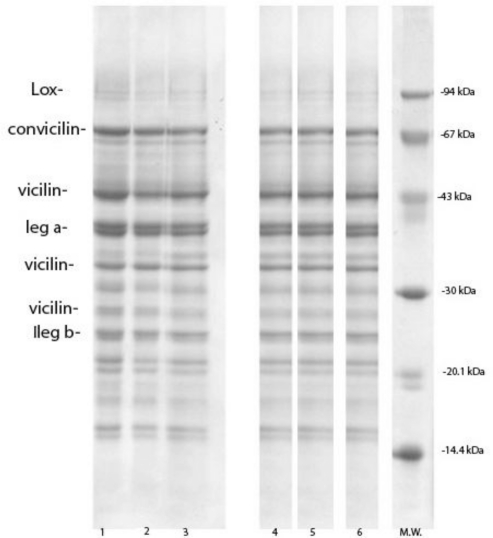
Electrophoretic patterns of pea protein isolates under reducing conditions. 1. L1; 2. L2; 3. L3; 4. Maja; 5. Calvedon; 6. Miracle of America; 7. M.w.-molecular weight markers.

In order to avoid a potential effect of other compounds such as sugars and polysaccharides on functional properties [39,40], pea proteins were isolated by isoelectric precipitation at pH 4.5. Protein precipitation retained most of 7S and 11S fraction and, as expected, pea protein isolates were their mixture (Figure 2). As a result of the precipitation, polypeptides of 9.8 kDa and 14.4 kDa disappeared almost completely, whereas protease inhibitor and lypoxigenase were reduced significantly. The concentration of PPI was 14.0 ± 0.70 g kg^−1^ to 26.2 ± 0.50 g kg^−1^, while concentration of lypoxigenase was 25.2 ± 0.2 g kg^−1^ to 43.5 ± 1.0 g kg^−1^ (Table 4). This was confirmed by SDS-electrophoretic and densytometric analysis of supernatants obtained after centrifugation and washing of precipitates. The concentration of protease inhibitors in supernatants was 229.2 ± 2.7 g kg^−1^ (Maja) to 349.1 ± 1.4 g kg^−1^ (L1), whereas the concentration of lypoxigenase ranged from 22.2 ± 0.4 g kg^−1^ to 64.4 ± 0.8 g kg^−1^ (Table 3). In addition, during pea isolates preparation, a part of subunits of vicilin of M.W. of 28.5 kDa, as well as a small part of hydrophilic α-subunits of legumin were lost. The concentration of these subunits in supernatants was ranged from 123.7 ± 2.4 g kg^−1^ to 173.3 ± 2.5 g kg^−1^ and 38.1 ± 0.2 g kg^−1^ to 58.9 ± 0.3 g kg^−1^ respectively (Table 3). After leaching and precipitation, the ratio of vicilin: legumin was reduced to 0.94 (Maja) and to 1.37 (L3), while the ratio vicilin + convicilin/legumin was 1.23–1.74.

### 3.4. Solubility

**Figure 3.**
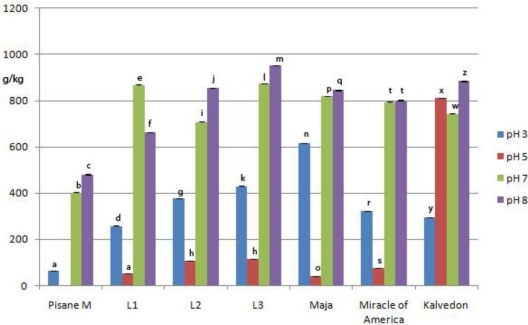
Solubility of pea protein isolate at different pH values *. * Bars with same letter differ (*p* < 0.05). Means were of triplicate determinations.

High solubility of proteins is desired for optimal functionality in food processing applications. Solubility of pea proteins of examined genotypes in aqueous solution was determined and compared to the solubility of commercial pea protein isolate. Figure 3 shows the change of solubility as a function of pH in the range of 3.0 to 8.0. Obtained solubility was significantly different (*p* < 0.01). Exceptions were the differences in solubility of genotypes L2 and Maja at pH 8.0 (855.8 g kg^−1^, 845.5 g kg^−1^). These results are similar to those reported for other legume proteins [25]. Isolates prepared from all investigated genotypes had significantly better solubility than commercial isolates at all pH values.

Pea proteins were almost insoluble at pH 5.0, but the solubility significantly increased below and above this pH value. At pH 3.0, solubility was ranged from 227.5 g kg^−1^ (L1) to 614.4 g kg^−1^ (Maja), whereas the solubility at pH 8.0 was 664.7 g kg^−1^ to 95.50 g kg^−1^. It is important to note that proteins from all investigated genotypes showed high solubility at pH 7.0 and 8.0. Also, high solubility was obtained for Maja at pH 3.0. Thus, all of them could be incorporated into products that have neutral or basic pH such as baked products or diet drinks, while Maja could also be useful for the preparation of products with low pH values.

### 3.5. Emulsifying Properties

**Figure 4.**
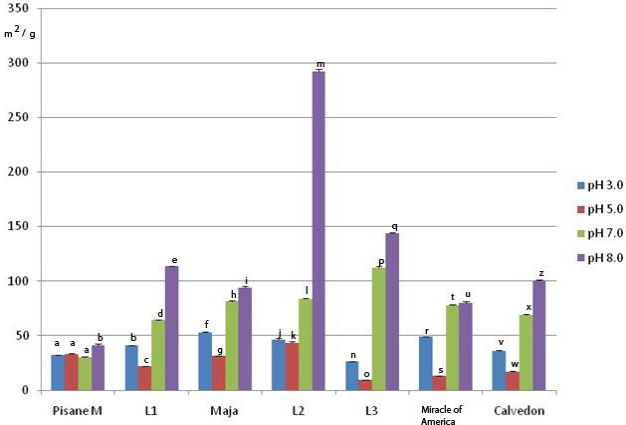
Emulsifying activity index (*EAI*) of pea protein isolates at different pH values *. * Within a parameter, bars with same letter differ significantly (*p* < 0.05). Means were of triplicate determinations.

**Figure 5.**
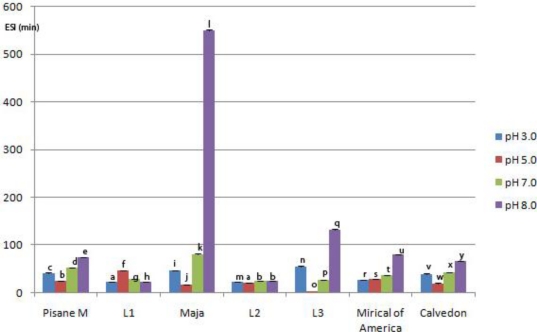
Emulsion stability index (*ESI*) of pea protein isolates at different pH values *. * Within a parameter, bars with same letter differ (*p* < 0.05). Means were of triplicate determinations.

Significant differences in *EAI* and *ESI* values were found among investigated genotypes (Figure 4 and Figure 5), as well as between emulsifying properties at different pH values of the same genotype. The exceptions were *ESI* values obtained for L1 and L2 at pH 3.0, and for L2 at pH 7.0 and 8.0. Furthermore, the emulsifying activity of pea proteins from investigated genotypes was significantly higher (*p* < 0.05) than the activity of commercial pea protein isolates when emulsions were formed at pH 3.0, 7.0 and 8.0. The highest *EAI* values was at pH 8.0 (93.92 ± 1.3 m^2^ g^−1^ to 291.94 ± 2.4 m^2^ g^−1^), whereas the lowest mean values for *EAI* were at pH 5.0 (9.27 ± 0.45 m^2^ g^−1^ to 31.63 ± 0.2 m^2^ g^−1^). The most unstable emulsion was prepared with proteins of L3 at pH 5.0 (1.5 ± 0.01 min), whereas the highest mean value of *ESI* was obtained at pH 8.0 with proteins of Maja (550 ± 2.20 min). No significant correlation was found between *EAI* and *ESI*. A strong positive correlation (0.80, *p* < 0.05) was found between solubility and *EAI*. This is in agreement with previous reports [22,26] which showed a positive relationship between protein solubility of pea or soybean proteins and emulsification capacity. Furthermore, our results suggested that there was no significant correlation between solubility and *ESI*.

Emulsifying ability of pea proteins was pH dependent. The lowest mean value of *EAI* was registered at pH 5.0 near I.e. of pea proteins, whereas emulsifying ability significantly increased below and above this value. Emulsifying ability was decreased at pH 5.0, probably as a result of increased protein-protein interactions and reduced solubility, which decreased flexibility and ability to form efficient interfacial membranes. On the other side, the commercial pea isolate was more resistant to changes in pH than the proteins of the laboratory prepared isolates. This is in agreement with a previous report of Aluko *et al.* [40]. Different resistance of laboratory prepared and commercial pea isolates to changes in pH, these authors explained by different processing history.

Generally, the highest emulsion stability of all pH values was obtained with proteins extracted from Maja, whereas the lowest mean values of *ESI* index were in L2. In contrast to *EAI*, a negligible influence of pH on emulsion stability of L2 was observed, whereas the best stability of emulsion formed with proteins from L1 was at pH 5.0. Proteins of the other genotypes formed more stable emulsions at the pH values above and below the I.e region. The highest stability of emulsion prepared with proteins of Maja, could be as a result of coactive effects of factors such as good solubility, protein composition and structure. This genotype had the lowest vicilin/legumin, as well as vicilin + convicilin/legumin ratios. It is known that these proteins have different structural properties. Legumin has more protein surface hydrophobicity or exposed hydrophobic groups than vicilin, which may lead to more adsorbed oil/protein on the interface. Also, in the pH range of 7–8, legumin partially dissociates into trimeric form. The dissociated form has less structured conformation and contributes to easier anchorage in the interfacial layer [41].

### 3.6. Foaming Properties

The ideal foam-forming and foam-stabilizing protein is characterized by a low molecular weight, high surface hydrophobicity, good solubility, a small net charge in terms of the pH of the food, and easy denaturability [42]. In general, the isolates prepared from investigated varieties are characterized by a significantly different (*p* < 0.01) foaming ability and low foam stability, regardless of the pH value of the suspension. No significant correlation was found between foaming properties and solubility while a moderate positive correlation (0.73) between *ESI* and *FC* was observed. This indicate that there are similar factors effecting foam ability and emulsion stability.

**Figure 7.**
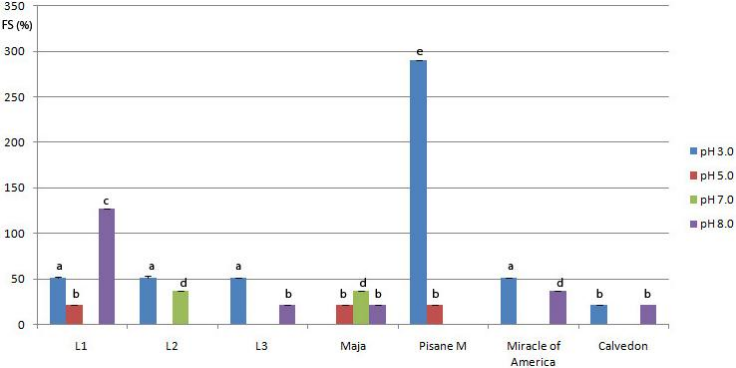
Foam stability (*FS*) of pea protein isolates at different pH values *. * Within a parameter, bars with same letter differ (*p* < 0.05). Means were of triplicate determinations.

The lowest *FC* and *FS* were obtained at pH 5.0. At higher pH (7.0, 8.0) foaming ability of laboratory prepared isolates increased. It seems that at higher pH, pea proteins had better (compared to pH 3.0 and 5.0) structural conformation, more suitable for interfacial membrane formation. Also, the results suggest that as the pH increases, the net charge density of proteins increases, which enhances protein unfolding and flexibility and contributes to better foam formation. The best foaming abillity was obtained with proteins of Maja (293.93%–439.39%). Except at pH 5.0, *FC*-values of Maja were significantly higher than those obtained for commercial pea isolate. Other genotypes had lower values of *FC* in relation to the commercial isolate. All examined isolates, except commercial isolate at pH 3.0 (290.40%) and isolates from L1 at pH 8.0 (127.30%) formed foam with low stability. For example, at pH 5.0 and 7.0, foam of isolates L3, Miracle of America and Calvedon completly dissapeared after 3 min, while at pH 3.0, *FC* of all isolates, except Maja, was only around 50% (Figure 7). Our results for foam stability differ from previous research repoted by Boye *et al.* [43]. They reported that pea isolates prepared by isoelectric precipitation formed stable foams. However, these contradictory results could be due to the different concentration of protein suspension used for analysis. In our research we used 1 g kg^−1^ protein suspension, wereas these authors prepred foam with more concentrated suspensions (5 times higher). The influence of protein concetration on foam stability was reported [40]. The stability of foam depends on the strength of the protein film and its permeability for gases. Film strength depends on the adsorbed amount of protein and the ability of adsorbed molecules to associate. At low concentrations, used in our research, either the amount of adsorbed proteins was too low, or high molecular weight proteins adsorbed on interface had low ability to associate.

## 4. Conclusions

The results of this study showed that a concentration of 7S and 11S was statistically different among the pea bean varieties. The ratio of these proteins has significant influence on pea protein extractability. Varieties with a higher level of 7S, and/or a lower level of 11S proteins, have higher extractability than the others. Solubility, emulsifying properties and foaming capacity of isolates prepared from investigated varieties were significantly different, and were pH dependent. On the other hand, low foam stability was obtained for all samples. The emulsion activity was strongly and positively correlated with the protein solubility. No significant correlation between solubility and emulsion stability or foaming properties was detected. Foaming capacity and emulsion stability was positively correlated. Our results suggest that genotype has influence on protein composition, as well as on technological-functional properties of pea proteins. This knowledge could be very useful in efforts to improve the quality of peas and pea protein products.

## Figures and Tables

**Figure 6.**
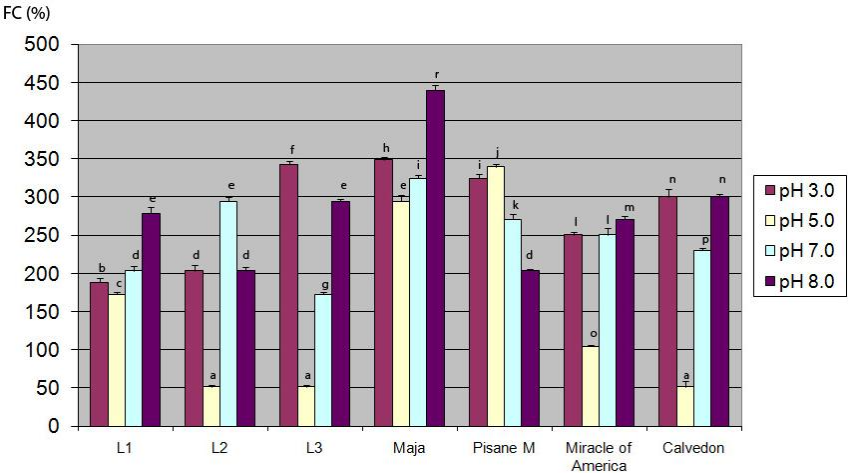
Foaming capacity (*FC*) of pea protein isolates at different pH values *. * Within a parameter, bars with same letter differ significantly (*p* < 0.05). Means were of triplicate determinations

**Table 1.** Content and extractability of pea bean proteins and protein content of pea isolates.

**Varieties**	**Total Protein^a^ Pea Bean (g/kg)**	**Extractable Protein Content^a^ (g/kg)**		**Extractability^b^**		**Total Protein^a^ Isolate (g/kg)**

		Tris-HCl pH 8.0	Water pH 8.0	Tris-HCl pH 8.0	Water pH 8.0	
Calvedon	244.21 ± 0.21^a^	147.81 ± 1.2^a^	107.42 ± 0.20^a^	605.2 ± 4.9^a^	439.8 ± 0.8^a^	837.71 ± 2.13^a^
L1	317.63 ± 0.29^b^	227.11 ± 0.62^b^	117.20 ± 1.01^b^	714.7 ± 1.9^b^	369.0 ± 3.1^b^	846.65 ± 1.54^b^
L2	241.42 ± 0.11^c^	142.20 ± 0.21^c^	97.61 ± 0.41^c^	589.0 ± 0.8^c^	404.3 ± 1.3^c^	835.09 ± 0.72^a^
L3	233.40 ± 0.3^d^	150.63 ± 0.82^a^	92.01 ± 0.6^d^	645.2 ± 3.4^d^	394.2 ± 2.1^d,g^	842.22 ± 0.67^a^
Maja	273.70 ± 0.10^e^	148.65 ± 1.43^a^	115.41 ± 0.8^b^	542.9 ± 3.4^e^	421.6 ± 2.8^e^	890.26 ± 2.47^b^
M.A.	223.31^f^	116,64 ± 0.22^e^	87.4 ± 0.4^e^	522.1 ± 0.9^f^	391.4 ± 1.8^f,g^	841.53 ± 1.09^a^

* Means followed by the same letter within the same column are not significantly different (*p* < 0.05);

a g of protein per kg of sample;

b g of protein per kg of protein; M.A. Miracle of America.

**Table 2.** Polypeptide composition of the investigated pea bean genotypes *.

**Protein**	**M.w.(kDa)**	**Concentration (g/kg)**					
		**Calvedon**	**L1**	**L2**	**L3**	**Maja**	**Miracle of America**
**Reduced Conditions**							
Lipoxygenase	92.7	25.5 ± 0.8^a^	48.1 ± 1.4^b^	25.9 ± 1.0^c,a^	29.4 ± 1.3^d,b^	24.5 ± 1.7^e,a,c^	17.4 ± 0.5^f^
Convicilin	77.9−72.4	83.8^a^	71.6 ± 2.0^b^	79.7 ± 0.6^c^	80.8 ± 1.7^d,c^	83.3 ± 1.4^a,d^	89.8 ± 2.2^e^
Vicilin	47.3	106.7 ± 1.1^a^	112.0 ± 1.0^b^	96.7 ± 2.0^c^	107.5 ± 0.2^d,a^	117.6 ± 0.7^e^	126.3 ± 0.3^f^
	37−31.8	159.6 ± 0.4^a^	181.9^b^	144.7 ± 1.7^c^	165.0 ± 0.7^d^	171.8 ± 0.4^e^	151.7 ± 1.2^f,c^
	28.7	75.6 ± 0.7^a^	83.4 ± 1.4^b^	103.5 ± 0.9^c^	102.6 ± 1.3^d,c^	79.7 ± 2.4^e,a^	89.3 ± 1.7^f,b^
		
Σ vicilin		341.9 ± 1.7^a^	377.3 ± 1.3^b^	344.9 ± 1.6^c,a^	375.1 ± 1.8^d,b^	369.1 ± 1.5^e^	367.8 ± 2.1^f,e^
Legumin α	40.89	122.1 ± 0.4^a^	80.4 ± 1.7^b^	86.3 ± 1.2^c^	98.4 ± 0.1^d^	151.2 ± 0.6^e^	102.8 ± 2.0^d^
Legumin β	23.1−22.3	129.7 ± 0.3^a^	96.4 ± 1.5^b^	125.8 ± 2.1^c,a^	127.2 ± 1.9^d,a,c^	157.6 ± 2.7^e^	166.1 ± 1.6^f^
Legumin_n.r_	63.6	33.6 ± 1.1^a^	75.6 ± 0.3^b^	51.6 ± 0.9^c^	55.5^d^	38.9 ± 1.0^e^	36.6 ± 0.2^f,e,a^
Σ legumin		285.4 ± 1.9^a^	252.4 ± 2.0^b^	264.7 ± 3.2^c^	281.1 ± 2.7^a^	347.7 ± 2.6^d^	305.5 ± 1.9^e^
V/L**		1.20 ± 0.0012^a^	1.49 ± 0.007^b^	1.30 ± 0.01^c^	1.33 ± 0.006^d^	1.06 ± 0.0036^e^	1.20 ± 0.0006^f^
V + C/L**		1.49 ± 0.0017^a^	1.78 ± 0.0017^b^	1.60 ± 0.014^c^	1.62 ± 0.003^d^	1.30 ± 0.0027^e^	1.50 ± 0.002^a^
Leg_n.r_/Leg_r_		0.13^a^	0.42^b^	0.24^c^	0.25^c^	0.13^e,a^	0.14^a,e^
PPI	11.5	7.73 ± 0.23^a^	6.67 ± 0.11^b^	7.55 ± 0.15^c,a^	7.05 ± 0.25^d^	8.02 ± 0.09^e^	10.81 ± 0.27^f^

**Non Reduced Conditions**							

lypoxigenase	92.7	31.3 ± 0.2^a^	43.3 ± 1.1^b^	35.3 ± 0.7^c^	31.5 ± 0.4^a^	40.1 ± 0.1^e^	27.3 ± 0.6^f^
PPI***	11.5	9.4 ± 0.1^a^	1.0^b^	9.0 ± 0.1^a^	6.2 ± 0.2^d^	3.6^e^	2.1 ± 0.3^f,b,e^
convicilin	77.9−72.4	112.0 ± 2.1^a^	112.5 ± 1.0^a^	118.5 ± 1.1^b^	114.4 ± 2.0^a,b^	123.4 ± 2.6^c^	215.1 ± 4.2^d^
vicilin	47.3	128.8 ± 0.9^a^	150.5 ± 2.3^b^	103.9 ± 0.7^c^	111.5 ± 1.5^d^	122.0 ± 1.1^e^	57.2 ± 1.4^f^
	37−31.8	189.1 ± 1.9^a^	178.2 ± 2.2^b^	170.8 ± 1.2^c^	188.5 ± 0.6^d,a^	197.6 ± 0.8^e^	220.2 ± 0.4^f^
	28.7	61.1 ± 0.2^a^	66.5 ± 1.0^b^	110.5 ± 0.3^c^	109.0 ± 1.3^c^	68.3 ± 2.4^e,b^	22.2 ± 0.9^f^
		
Σ vicilin		379.0 ± 3.0^a^	395.2 ± 5.5^b^	385.2 ± 2.2^c^	409.0 ± 3.4^d^	387.9 ± 4.3^b,c^	299.6 ± 2.7^f^
Legumin α	40.89	65.3 ± 1.0^a^	63.2 ± 2.7^b,a^	44.4 ± 0.1^c^	53.6 ± 2.2^d^	74.4 ± 1.2^e^	42.4 ± 1.1^f,c^
Legumin β	23.1−22.3	72.8 ± 0.8^a^	58.0 ± 1.4^b^	59.0 ± 1.9^c,b^	56.8 ± 0.3^d,b,c^	73.0 ± 2.0^e^	61.8 ± 0.7^f,c^
Leg_n.r._	63.6	152.7 ± 2.5^a^	75.4 ± 1.1^b^	122.5 ± 1.6^c^	106.6 ± 1.5^d^	155.8 ± 0.9^e,a^	127.5 ± 1.5^f,c^
		
Σ Leg		290.8 ± 4.3^a^	196.6 ± 5.1^b^	225.9 ± 3.6^c^	217.0 ± 4.0^d^	303.2 ± 4.1^e^	231.7 ± 2.6^c^
Legumin α	40.89	65.3 ± 1.0^a^	63.2 ± 2.7^b,a^	44.4 ± 0.1^c^	53.6 ± 2.2^d^	74.4 ± 1.2^e^	42.4 ± 1.1^f,c^
V/L		1.30 ± 0.009^a^	2.09 ± 0.026^b^	1.70 ± 0.017^c^	1.88 ± 0.019^d^	1.28 ±0.022^e^	1.29 ± 0.0027^a^
C + V/L		1.69 ± 0.031^a^	2.54 ± 0.036^b^	2.23 ± 0.021^c^	2.41 ± 0.019^d^	1.69 ± 0.021^a^	2.22 ± 0.005^c^
Leg_n.r_/Leg_r_		1.11 ± 0.03^a^	0.62^b^	1.18 ± 0,02^a^	0.97 ± 0.04^d^	1.06 ± 0.07^a^	1.22^c^

* Means followed by the same letter within the same row are not significantly different (*p* < 0.05). Means were of triplicate determinations;

** V/L-vicilin to legumin ratio; V + C/L-vicilin + convicilin to legumin ratio;

**** PPI-pea protease inhibitor.

**Table 3.** Protein composition of the investigated pea isolates.

**Protein/polypeptide**	**M.w.(kDa)**	**Concentration (g/kg)**					
		**Calvedon**	**L1**	**L2**	**L3**	**Maja**	**Miracle of America**
**Isolate**							

lipoxygenase	92.7	29.8 ± 0.7^a^	25.2 ± 0.2^b^	34.3 ± 0.4^c^	43.5 ± 1.0^d^	36.8 ± 1.4^c^	27.7 ± 0.5^a^
convicilin	77.9−72.4	104.2 ± 0.1^a^	102.7 ± 1.1^a^	117.8 ± 2.6^b^	103.8 ± 1.6^a^	91.3 ± 0.6^c^	97.4 ± 1.2^d^
Vicilin	47.3	153.1 ± 4.3^a^	171.8 ± 0.9^b^	168.3 ± 2.7^b,c^	176.8 ± 3.4^d^	123.0 ± 1.1^e^	166.7 ± 2.4^b^
Vicilin	37−31.8	195.6 ± 1.7^a^	207.0 ± 3.3^b^	194.1 ± 3.1^a^	207.7 ± 2.2^b^	177.5 ± 3.6^c^	188.7 ± 1.8^d^
Σ vicilin		348.7 ± 6.0^e^	378.8 ± 4.2^a^	362.4 ± 5.8^b^	384.5 ± 5.0^c^	300.5 ± 4.7^d^	355.4 ± 4.2^f,b^
legumin α	40.89	133.5 ± 2.6^a^	162.3 ± 2.1^b^	155.6 ± 3.9^c^	95.6 ± 2.9^d^	115.3 ± 1.1^e^	142.0 ± 0.7^f^
Legumin β	23.1−22.3	144.2 ± 0.2^a^	142.7 ± 0.7^b^	142.0 ± 0.4^b^	152.8 ± 2.2^c^	168.2 ± 1.7^d^	147.0 ± 0.3^,e^
Leg_n.r_	63.6	35.4 ± 0.7^a^	24.6 ± 0.2^b^	36.8 ± 0.5^a^	30.8 ± 0.4^c^	34.3 ± 2.1^a^	32.7 ± 0.4^f,c,d,e^
Σ legumin		313.1 ± 3.5^a^	329.6 ± 3.3^b^	334.4 ± 5.0^b^	280.2 ± 4.9^c^	317.8 ± 4.9^,da^	321.7 ± 1.4^d^
V/L		1.11 ± 0.0067^a^	1.15 ± 0.0018^b^	1.08 ± 0.0011^c^	1.37 ± 0.0061^c^	0.94 ± 0.0002^d^	1.10 ± 0.0082^c^
C+V/L		1.45 ± 0.003^a^	1.46 ± 0.002^b^	1.43 ± 0.0036^c^	1.74 ± 0.0069^d^	1.23 ± 0.0023^e^	1.41 ± 0.01066^f^
PPI		17.9 ± 0.8^a^	17.0 ± 0.2^a^	26.2 ± 0.5^c^	17.6 ± 0.5^a^	14.0 ± 0.7^d^	21.0^f^
**whey**							
lipoxygenase	92.7	33.7 ± 0.9^a^	22.2 ± 0.4^b^	26.2 ± 0.2^c^	29.8^d^	64.4 ± 0.8^e^	32.4 ± 0.8^a^
Legumin α	40.89	45.20 ± 0.4^a^	58.9 ± 0.3^b^	50.1 ± 0.1^c^	54.9 ± 0.7^d^	38.1 ± 0.2^e^	47.1 ± 0.4^f^
vicilin	28.5	154.2 ± 1.1^a^	131.0 ± 1.0^b^	168.7 ± 1.2^c^	173.3 ± 2.5^d^	123.7 ± 2.4^e^	140.2 ± 2.1^f^
PPI	11.5	261.1 ± 1.7^a^	349.1 ± 1.4^b^	242.0 ± 2.20^c^	324.6 ± 2.7^d^	229.2 ± 2.7^e^	253.3 ± 3.7^f^

* Means followed by the same letter within the same row are not significantly different (*p* < 0.05). Means were of triplicate determinations;

** V/L-vicilin to legumin ratio; V + C/L-vicilin + convicilin to legumin ratio;

**** PPI-pea protease inhibitor.
